# The variation in promoter sequences of the *Akt3* gene between cow and buffalo revealed different responses against mastitis

**DOI:** 10.1186/s43141-021-00258-4

**Published:** 2021-10-22

**Authors:** Farmanullah Farmanullah, Mostafa Gouda, Zhang Min, Xu Sutong, Mohib Ullah KaKar, Sami Ullah Khan, Muhammad Salim, Momen Khan, Zia ur Rehman, Hira Sajjad Talpur, Faheem Ahmed Khan, Nuruliarizki Shinta Pandupuspitasari, Zhang Shujun

**Affiliations:** 1 Faculty of Veterinary and Animal Sciences, National Center for Livestock Breeding Genetics and Genomics LUAWMS, Uthal, Balochistan Pakistan; 2 Key Laboratory of Animal Genetics, Breeding and Reproduction, Ministry of Education, College of Animal Science and Technology, Huazhong Agricultural University, Wuhan, 430070 Hubei China; 3 College of Biosystems Engineering and Food Science, Zhejiang University, 866 Yuhangtang Road, Hangzhou, 310058 China; 4 Department of Nutrition & Food Science, National Research Centre, Dokki, Giza, 12622 Egypt; 5 Faculty of Marine Sciences, Lasbela University of Agriculture Water and Marine Sciences (LUAWMS), Uthal, Balochistan 90150 Pakistan; 6 Department of Internal Medicine, Faculty of Veterinary Sciences, University of Gadjah Mada, Yogyakarta, Indonesia; 7 Department of Forestry and Wildlife Management, The University of Haripur, Khyber Pakhtunkhwa, Pakistan; 8 Directorate General (Extension) Livestock and Dairy Development Department, Bacha Khan Chowk, Peshawar, Khyber Pakhtunkhwa Pakistan; 9 Department of Animal Health, Faculty of Animal Husbandry and Veterinary Sciences, The University of Agriculture Peshawar, Peshawar, Pakistan; 10 Department of Animal Breeding and Genetics, Sindh Agriculture University Tandojam, Hyderabad, Pakistan; 11 Laboratory of Molecular Biology and Genomics, Department of Zoology University of Central Punjab, Lahore, Pakistan; 12 Laboratory of Food Biotechnology, Faculty of Animal and Plant Science, Diponegoro University, Semarang, Indonesia

**Keywords:** *AKT3*, Promoter, pGL3, mRNA, Expression, LPS, Immune response

## Abstract

**Background:**

Serine/threonine kinase 3 (*AKT3*) is a protein-coding gene that is associated with several cattle immune diseases including different tumors and cancers. The objective of this study was to investigate the differences in structures and functions of *AKT3* of cow and buffalo cattle.

**Methods:**

The sequence differences of gene-coding sequence (CDS) and core promoter region of *AKT3* in cow and buffalo were analyzed by using bioinformatics tools and PCR sequencing. Also, the functional analysis of promoter regulating gene expression by RT-PCR was performed using 500 Holstein cows and buffalos. And, evaluation of *AKT3* inflammatory response to the lipopolysaccharide (LPS)-induced mastitis was performed between both species.

**Results:**

The results revealed the variation in 6 exons out of 13 exons of the two species of CDS. Also, 4 different regions in 3-kb promoters of the *AKT3* gene were significantly different between cow and buffalo species, in which cow’s *AKT3* promoter sequence region was started from − 371 to − 1247, while in buffalo, the sequence was started from − 371 to − 969 of the promoter crucial region. Thus, the promoter was overexpressed in cows compared to buffaloes. As a result, significant differences (*P* < 0.05) between the two species in the *AKT3 gene* expression level related to the LPS stimulation in their mammary epithelial cell line.

**Conclusions:**

This study emphasized the great importance of the structural differences of *AKT3* between the animal species on their different responses against immune diseases like mastitis.

## Background

The livestock genetic diseases’ impact on its global production and security showed exceptional importance during the last few decades, which significantly affects human civilization, development, and world essential protein consumptions [1].

Most of the diseases that affect cattle production have a relationship with their immune genes, in which Xie and Weiskirchen [2] reported that *AKT3* stands for serine/threonine-protein kinase-encoded oncogene. The importance of these genes in the diagnosis and treatment of different cattle diseases lets scientists try to deeply investigate these genes and their functions. For instance, mastitis disease is one of the *AKT3-*related infections in dairy cattle which caused a massive problem and a significant negative impact on the global dairy economy. The researchers have done a lot of work to prevent, control, and treat mastitis disease and reported mastitis and dairy farming; however, it is still a remarkable issue in dairy farm management. The somatic cell count and somatic cell score play role in association with mastitis and the phenotypic data is required for the association study [3] and mastitis association with somatic cell count study is still under exploration, and further, genetic risk factors and other essentials of mastitis are needed to be addressed [4].

Beecher et al. [5] reported that the relationship of mastitis with SNPs and genes is important based on the vital role of genes in mammary gland immune response for regulating mastitis. Several diverse research tools are using for advanced research in the field of investigation and association with disease, such as the gene mapping in genetics with genomic chips, and recent bioinformatics tools for candidate genes were approached. The important economic traits in dairy animals and the sequence of genes on quantitative trait locus association study and analysis are very important. It has been established as an effective tool for assisting in marker selection against these diseases of economically significant traits. Pimentel et al. [6] reported that for vital traits such as reproduction, immunity, and milk production, single nucleotide polymorphisms were used for candidate gene approach and selection. The association and gene expression for *AKT3* with mastitis and its role in immune response as well as the sequence differences between cow and buffalo has not been reported yet. The transcription initiation and gene regulation are very important for gene expression and are controlled by the promoter area. Promoters in upstream of a gene with transcription start site (TSS) is very important for various functions and binding of RNA polymerase [7] and the promoter identification, as well as important consensus sequences, core promoters, and their analysis for the promoter.

YJ Mao et al. [8] reported that the CpG island is also one of the important factors which occur adjacent to the core promoter in humans with 50% probability, in which many model algorithms and computational analyses have been used for promoter analysis and prediction, while the TSS and CpG island and core promoter study for the promoter are usually understood. L Zhang et al. [9] reported that gene expression and physiology are also correlated with various components, which play a very crucial role in gene expressions such as genetic change cis-regulatory and coding sequence if it is compared to various species of specific phenotypes. The new insight in molecular level between cow and buffalo naturally for promoter prediction, differences in genetic sequences, in the upstream and 5′ UTR for the candidate gene could be imperative for gene expression, binding sites, and alteration in transcription. Besides, the cow and buffalo are different but belong to the same origin and phylogeny, but cows are more susceptible to disease as compared to buffalo with mastitis. Khanal and Pandit [10] reported that the prevalence of mastitis in cows is highly predominant than in buffaloes; although they are of the same origin, the cow is more susceptible to mastitis disease. Understanding this mechanism and the fact of genetic analysis show the differences of cow and buffalo sequences for mastitis and ensure the limits as previously no such study is performed for cow and buffalo. Therefore, it is noteworthy to study this candidate gene of *AKT3*, as they are pro-inflammatory interplays of signaling pathways, and it would be vital for the future as well currently for promoter differences and variation inclusive study in cow and buffalo sequences.

Driessche et al. [11] described that transcription measurement can identify, characterize, and classify the core promoter in the gene sequence, in which RNA polymerase II and III have the potential to cluster both infected and healthy cells. Additionally, the epigenetic study is important for correlating the genes’ functionality with physiological phenotyping [12]. Furthermore, promoter, chromatin remodeling, methylation, and histone modification studies are considered the most important epigenetic studies of the DNA, in which the importance of the plasmid-basic-luciferase vector 3 (pGL3) is for the ligation and cloning of the specific target gene like *AKT3* through the use of specific restriction enzymes capable with specific restriction sites like Kpnl and BgIII [13]. That can amplify and detect the upstream region of the gene concerned with the promoter, core promoter, and other important consensus sequences which play an important role in the modification of a gene, transcription binding sites, and its post-translation and expression. Therefore, the proportional, variation, and comparison of promoter studies in cow and buffalo sequence differences could affect their responses to mastitis disease performance.

Thus, the objective of this study was to find out the differences, association, and expression for *AKT3* in cows and buffaloes. The bioinformatics tools and PCR sequencing were used and correlated to verify the functionality of promoter gene expression performance. This knowledge might help to provide a target for intervening against the different responses of mastitis disease in cows and buffalos.

## Methods

### Animals

A total number of 500 Holstein cows (244) and buffalos (256) were used in this study from Hubei province of the dairy herd, Hubei, China. Also, this study sampling was carried out in the Hubei province of the dairy herd, China. The weather was sub-humid tropical climates with annual rainfalls of 1100–1200 mm. The average temperature was 16–17 °C; relative humidity was 75%, in which mainly cows and buffalos were practiced free stall barns with total mixed ration (TMR) three times daily.

### Animal blood sampling and extraction of genomic DNA

Blood samples were collected in a tube with EDTA (pH = 8.0) anticoagulants from Holstein cows and buffalos. Also, blood samples with good Danhong injection (DHI) were collected from animals; no further treatments or uses of the animals under the study were made. And the animals were still alive and in good health after the study.

**Fig. 1:**
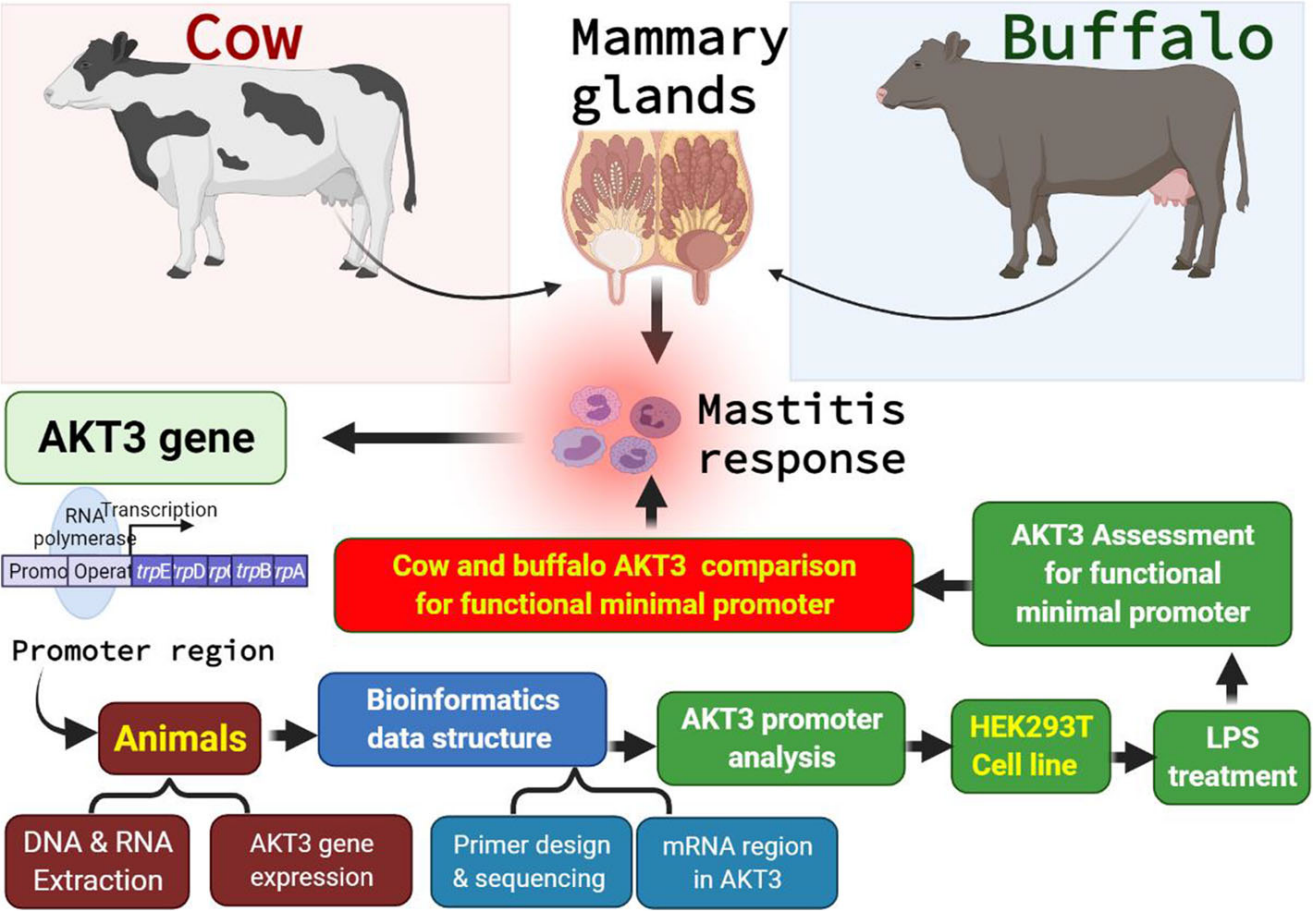
Flow chart of the experimental design

Before genomic DNA extraction, the samples were kept at − 20 °C. Wang et al.’s [14] protocols were used for DNA extraction. A NanoDrop^TM^ spectrophotometer (ND-2000c, Thermo Scientific, Huston, USA) was used to measure the quantity and quality (260/280 nm) of the isolated genomic DNA. A flow chart for the entire experimental design is presented in Fig. 1.

### Bioinformatics data structure

The various gene banks Ensemble, NCBI, and Uniprot of public databases were used for the data sequence of the *AKT3* gene for cow and buffalo (http://useast.ensembl.org/index.html), (www.ncbi.nlm.nih.gov/genbank), (http://www.uniprot.org). The *AKT3* gene sequence of buffalo and cattle were (NW-005783781.1) and (AC-000173.1) that were selected from NCBI and Ensemble. The bioinformatics approach was used to target the *Akt3* gene differences between cow and buffalo for various paramount regions [15].

### Primer design and sequencing

The *AKT3* gene sequences of buffalo (NW_005783781.1) and cattle (AC_000173.1) were selected from the database of the National Centre for Biotechnology Information (NCBI https://www.ncbi.nlm.nih.gov/gene/?term=*Akt3*) [15]. The NCBI database was used for the annotation of the *AKT3* gene of cows and buffaloes. The protocol was used by the reported for mutation of SNPs region as DNA pooling methods [16]. We have design primers for various regions of the target fragment to amplify that exon and adjacent intronic region of cow and buffalo. The Primer Premier 5.0 software (Premier Biosoft International, Palo Alto, CA, USA) was used to design primers for the specific region and fragment for amplification of the *AKT3* in cow and buffalo, and the primers were synthesized by Sangon Biological Technology (Shanghai, China). The 25 μl total volume mixture contained genomic DNA, reaction mix, each primer, and taq DNA polymerase (including 500 μM dNTP each; 20 mM Tris-HCL; pH 9; 100 mM KCl; 3 Mm MgCl_2_), with the ratio 50 ng, 12.5 μl 2 X, 0.5 μM, and 0.5 units, respectively. The protocol for cycling was followed as 95 °C for 5 min, denaturing cycles as 94 °C for 30 s, annealing as 30-s temperature (Tm), extending at 30 s for 72 °C, with a final extension of 10 min at 72 °C.

### Differences in cow and buffalo for CDS/mRNA region in *AKT3*

The main focus and objective of this study were to find out the differences and variations of the *AKT3* for cows and buffaloes. There are thirteen exons in cow and buffalo *AKT3*; we were interested to find out the variation, differences in the cow and buffalo for coding sequence, mRNA, and confirmation experimentally. The susceptibility of cows to mastitis and other diseases is higher than buffalo. Therefore, the recent project planned to carry out the differences, we blasting exon wise, one by one comparing and checking the similarity and differences through NCBI blasting tool the exon sequences, mRNA of cow and buffalo, and further experimentally work and confirmation. For the sequences similarity, differences and mRNA analysis performed the highly similar sequence (mega blast). NCBI blast tools were used (http://blast.ncbi.nlm.nih.gov/Blast.cgi).

### Approach to promoter analysis and bioinformatics

For predicting the transcription start sites, in buffalo and cow, and the promoter sequence prediction, a bioinformatics tool was performed by using http://www.fruitfly.org/seq tools/promoter.html [17]. The CpG islands in buffalo and cow were predicted using MethPrimer (http://www.urogene.org/methprimer/) [18] and the tools provided at http://www.ebi.ac.uk/Tools/emboss/cpgplot/ [19, 20].

### Primers construction for cow and buffalo promoters

Various primers for the upstream gene promoters region were prepared and characterized for the different regions of the upstream through the Primer Premier 5.0 software (PREMIER Biosoft Int., CA, USA).

### Culture and LPS treatment of the bovine epithelium cell

The HEK 293T (human embryonic kidney 293T) cell line was used to measure the mRNA expression level of the *AKT3*. The standard protocol of HEK 293T following cell culture used a Roswell Park Memorial Institute (RPMI) 1640 medium (Thermo Fisher, Massachusetts, USA) which contains bovine fetal serum, non-essential amino acids, penicillin, and streptomycin with the ratio of 10%, 1%, 100 U/ml, and 100 mg/ml, respectively. And that is useful for high-efficiency transfection and expression of genes [21]. The cell culture was maintained at 37 °C and 5% CO_2_. GIBCO reagents (Gibco, Grand Island, NY, USA) were used for this experiment. The LPS dose in the treated cells was standardized in our lab already, which is used as 10.0 ng/ml. The cells were plated with a good density of 1.0 × 10^5^ cells/well in 24-well plates, and after 24 h, 10 ng/ml of LPS was added into the cells. Culture media were used to seed cells at 85–90% confluence and without antibiotics; culture media used 10% FBS and cultured overnight at 37 °C. Three replicates were made after the LPS treatment at various times (3 h, 6 h, 12 h, and 24 h, respectively) for RNA extraction.

### RNA extraction, cDNA, and QPCR

Different kits are available for various procedures and processing; Trizol kit was for total RNA extraction (Invitrogen, Carlsbad, CA) following the instruction and steps, while 1× HBSS was used twice to rinse the culture cells. Table 1 presents the QPCR experiment steps. The Nanodrop spectrophotometer absorbance was used for the purity and quantity of RNA, with 260/280 nm of optical density. The cDNA strand was synthesized using a cDNA kit (Toyobo Co., Japan). The Primer Premier 5.0 software was used for the respective gene primer design and preparation and confirmed through the bioinformatics UCSC software. QPCR implication was performed (Bio-Rad iQ5 real-time PCR system, CA, USA); master mixes, RNA-free water, and primers were the ingredients of the reaction master mixture. In addition, there was a control group in the experiment after normalization of mRNA level and a control group of actin and RNAse free water. Also, the experiment samples were conducted in three replicates.

**Table 1:** presents the QPCR experiment steps

No.	Step process
1	Total RNA extraction.
2	Confirm the good purity and quantity of RNA, with 260/280 nm > 1.8 and >200 ng yield by NanoDrop spectrophotometer.
3	cDNA strand Synthation using cDNA kit
4	QPCR primer design using premier 5.0 for respective genes and confirmation of their accuracy through bioinformatics UCSC software.
5	The QPCR of the cDNA was performed by using master mix probe and the previously designed and checked primers. The protocol was as follows: 95 °C for 5 min, denaturing cycles as 94 °C for 30 s, annealing as 30-s temperature (Tm), extending at 30 s for 72 °C, with a final extension of 10 min at 72 °C.
6	Control group in experiment as after normalization of mRNA level and as a control group of actin.
7	The experiment samples were conducted in three replicates.

### Cloning of promoter and generation of luciferase reporter constructs

Specific primers were designed to amplify that specific upstream region of the 2.0-kb genomic region with other substantial sequences. KpnI and BgIII (TaKaRa, Dalian, China) were used for the excision and digestion of the 2.0-kb fragment of the promoter in bovine *AKT3*. It was used for the generation of the luciferase construct, as the PGL3 basic vector was used for ligation and digestion with the same restriction enzymes. This plasmid is known as PGL3-1933, while other plasmids were characterized according to their size and length, which includes unidirectional deletions of the promoter, and by using specific primers, it is produced by PCR. The PGL3-1933 was used as a template using specific primers with restriction of incorporated Kpnl and BgIII restriction sites. Quick Change Site-Directed Mutagenesis Kit (Stratagene, La Jolla, CA, USA) was used for the construction of substitution mutation and regulation; it was sequence made and constructed in both directions.

### Transient transfection and luciferase reporter assay

The 48-well plates with a density of 1.2 × 10^5^ cells/wells were used for the transfection. After 24 h of reaching the density, they were transfected with the vector plasmids. With the help of the Lipofectamine 2000 kit (Invitrogen), 450 ng was added to each well of the cell plate for the transfection process. A PGL3 basic vector was used as a negative control. We have also used the same recombinant plasmid value of 450 ng for the 50-ng pRL-TK as co-transfected. The transfected plates were inoculated overnight at 37 °C, 85–90% humidity, and 10% FBS. The time and duration of the protocol for each step are very important and should be cared for properly. The phosphate-buffered saline was used for cell washing after the transfection phase. For the preparation of both Renilla and Firefly cell lysates, a passive lysis buffer was used for preparing the total lysates according to the Promega kit instruction (Promega, NC, USA). Also, the independent experiments and ratio for both Firefly and Renilla luciferase light units were analyzed.

### The assessment, association, evaluation, and comparison of the cow and buffalo *AKT3* for functional minimal promoter

The 293T cells were used for testing and checking the level of strength of promoter fragment construct vectors in cow and buffalo. The 293T cells were used for the transfection of the luciferase gene after 48 h, and for the luciferase assay, the cells were harvested. The activities of the relative luciferase of the promoter fragments were represented by bars. Primers were used for mRNA of the *AKT3* gene. The list of the primers used is listed in Table 2.

**Table 2:** List of primers used for mRNA expression

No.	Primers	Temperature
1	AGGTTGGGTTCAGAAGAGGG AAGTTGTTGAGGGGATAAGG	58.0
2	TTGTGAAAGAAGGTTGGGTT CTGAAAAGTTGTTGAGGGGATA	59.0
3	GAAAAATATGATGAGGACGG TGTAAGAGTTAGGACTGCTGTG	60.0

### Statistical analysis

Different research and statistical model were used for various types of group data. We used it for the detection and to find out the significant result of the group data. One-way ANOVA supporting unpaired *t*-test was used for the group data analysis to detect significant differences (*p* < 0.05) of the group data which presented as a whole the experimental data. The values were described as mean ± SD, and various statistical significance levels of the experimental group data three times independently were represented as *p < 0.05, **p < 0.01, and ***p < 0.001.

## Results

### Differences and functions of CDs in the *AKT3* gene between cow and buffalo using bioinformatics analysis

The main objectives of this part were to investigate the differences and their function of the CD region between cow and buffalo and their association study with mastitis. The targeted regions were CDs and important core promoter regions of cow and buffalo.

The detailed gene structure data of various parts of the *AKT3* gene reported in cows and buffalo are presented in Table 3. There was a total of 13 exons in cows and buffaloes for the *AKT3* gene. The high similarity sequence (mega blast) analysis of the exon was performed between cow and buffalo using the NCBI blast tool (http://blast.ncbi.nlm.nih.gov/Blast.cgi). After comparing, blasting of 13 exon sequences was found with substantial differences in 6 out of 13 exons, 4, 6, 7, 9, 12, and 13; each of the first 5 exons has one nucleotide difference while exon 13 has four nucleotide differences in its sequences between cow and buffalo (Tables 3 and 4).

**Table 3:** Features of parts of *AKT3* length by base pair (bp) for cow and buffalo

Species	Exon no.	Exons	Coding region	Exon (bp)	Coding (bp)	Intron (bp)	T. annotated spliced exon	Annotated AA (bp)	***AKT3*** (bp)
**Cow**	1	34230549–34230635	34230590–34230635	87	46	128152	1776	479	
	2	34358788–34358913	34358788–34358913	126	126	30063			
	3	34388977–34389088	34388977–34389088	112	112	5598			
	4	34394687–34394831	34394687–34394831	145	145	13332			
	5	34408164–34408295	34408164–34408295	132	132	7969			
	6	34416265–34416330	34416265–34416330	66	66	457			
	7	34416788–34416856	34416788–34416856	69	69	24346			
	8	34441203–34441325	34441203–34441325	123	123	7114			
	9	34448440–34448568	34448440–34448568	129	129	10409			
	10	34458978–34459192	34458978–34459192	215	215	7157			
	11	34466350–34466437	34466350–34466437	88	88	24704			
	12	34491142–34491244	34491142–34491244	103	103	6788			
	13	34498033–34498413	34498033–34498118	381	86	Nil			
*Total L*	*13*	*1.8 kb*	*1.4 kb*	*1.8 kb*	*1.4 kb*	*263.3 kb*			*265.1 kb*
**Buffalo**	1	627333–627348	Nil	16	Nil	10300	6517	437	
	2	637649–637809	637764–637809	161	46	162263			
	3	800073–800184	800073–800184	112	112	5649			
	4	805834–805978	805834–805978	145	145	12036			
	5	818015–818146	818015–818146	132	132	8245			
	6	826392–826457	826392–826457	66	66	456			
	7	826914–826982	826914–826982	69	69	23425			
	8	850408–850530	850408–850530	123	123	7137			
	9	857668–857796	857668–857796	129	129	10278			
	10	868075–868289	868075–868289	215	215	7344			
	11	875634–875721	875634–875721	88	88	25533			
	12	901255–901357	901255–901357	103	103	6899			
	13	908257-913414	908257-908342	5158	86	Nil			
*Total L*	*13*	*6.5 kb*	*1.3 kb*	*6.5 kb*	*1.3 kb*	*279.6 kb*			*286.8 kb*

**Table 4:** List of nucleotides differences between cow and buffalo in 6 exons

No.	Exon	Variation/mutation	mRNA position in cow	AA position in cow sequence	AA changed
		Cow/buffalo			
1	4	A/G	365	122	D/G
2	6	C/T	617	206	T/I
3	7	A/G	704	235	Y/C
4	9	A/G	971	324	Q/R
5	12	A/G	1322	Yes441	H/R
6	13	G/C	1593	Yes531	P/P
7		T/C	1617	Yes539	P/P
8		G/A	1623	Yes541	P/P
9		T/C	1643	Yes548	I/T

### The *AKT3* characterization of 5′ UTR regulatory region in cow and buffalo based on bioinformatics

The *AKT3* characterization of the 5′ UTR regulatory region was used for prediction of promoter and confirmation of the transcription start site (TSS) position in cow and buffalo *AKT3*. We characterized the 3.0-kb upstream of the *AKT3* in cow and buffalo which are related to mastitis inflammation (Table 5). Core promoters (TSS/TATA) was 2/7 for cows compared to 2/4 for buffalos which could be one of the substantial elements that play a central role in the regulatory function of gene and that could be the reason for the higher susceptibility of cows than buffalos to mastitis disease.

**Table 5:** List of predicted promoters and other important sequences in upstream of the *AKT3* In cow and buffalo

No.	Predicted promoters	Score	Range	Core promoters (TSS/TATA)		GC box	CpG island	Caat box	Species
**1**	ACCTCAGTGTCAAAAATCTGCCTACTGCTATCTCTGCCTCAAAAACAAAG	0.86	359–409	Two	Seven	No	No	No	**Cow**
**2**	AAAAAATCTACATATAAGTGGACCTGCACAGTTCAAACCCATGTTGTTCA	0.98	589–639						
**3**	ACGAATTGTATTTAAATCTGGGAATCAGCTTAATCTCTGTAAGCCTCAAT	0.95	2019–2069						
**4**	AACTACTTAATAAATAGGTTCCGCAGTTTGAAGAGCTAGACTTTTATGTG	0.89	2620–2670						
**5**	TGTCCCTTGTTATAAGTATGGCCAAAGAAGGTCTAAGTGCAGTTCTTCTA	0.81	291–341	Two	Four	No	No	No	**Buffalo**
**6**	ACCTCAGTGTCAAAAATCTGCCTACTGCCATCTCTGCCTCAAAAACAAAG	0.90	427–477						
**7**	AAAAAATCTACATATAAGTGGACCTGCACAGTTCAAACCCATGTTTGTTG	0.98	659–709						
**8**	ACTAATTGTATTTAAATCTGGGAATCAGCTTAATCTCTGTAAGCCTCAAT	0.95	2092–2142						
**9**	AACTACTTAATAAATAGGTTCCGCAGTTTGAAGAGCTAGACTTTTATGTG	0.89	2690–2740						

**Fig. 2:**
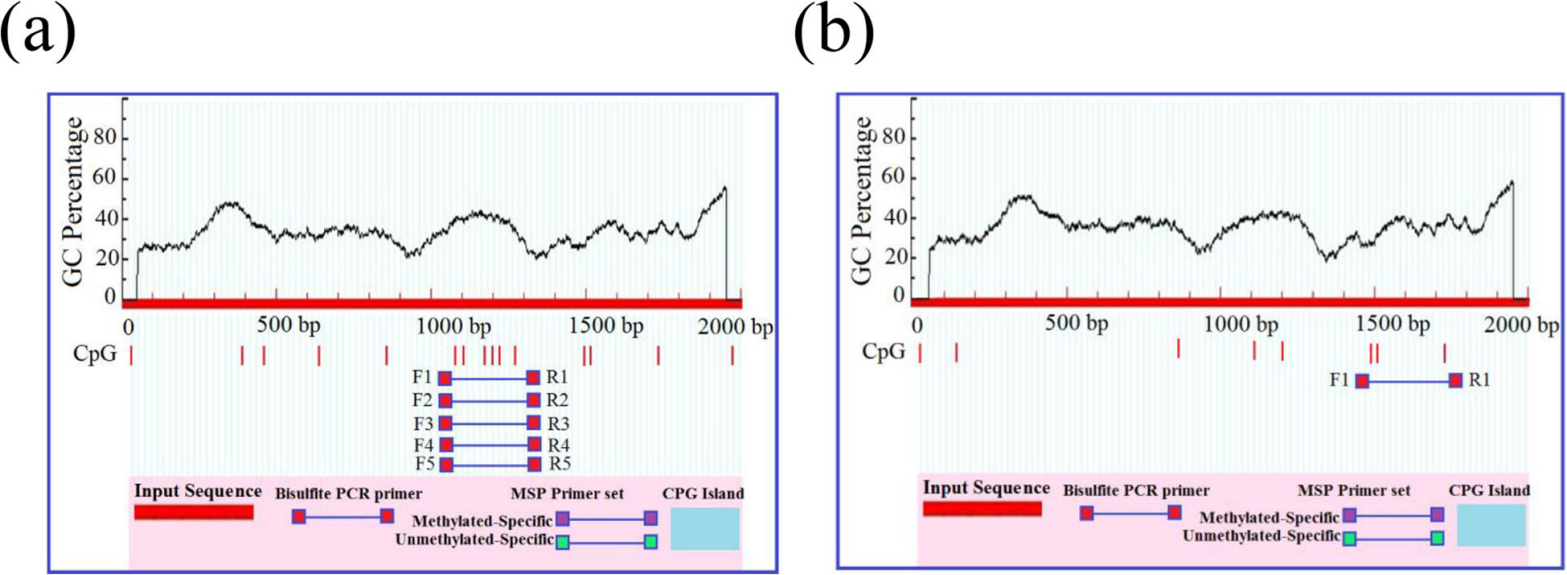
**a** Cow sequence length 3.0 kb, CpG island prediction result, no CpG island were found, criteria used: island size > 100, GC percent greater > 50.0, Obs/Exp > 0.6. **b** Buffalo sequence length 3.0 kb, CpG island prediction result, no CpG island were found, criteria used: island size > 100, GC percent greater > 50.0, Obs/Exp > 0.6

Further, the expression level of these various promoter regions in cow and buffalo was studied. For this experiment, we have used the − 3000-bp upstream of the *AKT3* sequences in cow and buffalo considering ATG as +1, and to predict various regions of the possible promoters, TSS, and other important sequences in the upstream of cow and buffalo. The predicted possible promoter regions were reported as with the distance from starting ATG of the *AKT3* in cow were − 225 to − 275, − 826 to − 876, − 2256 to − 2306, and − 2486 to − 2536. The CpG island methylation and possible transcription activity sites are correlated with each other, as the CpG island is necessary for the conceivable activity sites of transcription. However, no CpG island was found in the upstream region of cow and buffalo for the gene under study (Fig. 2).

**Fig. 3:**
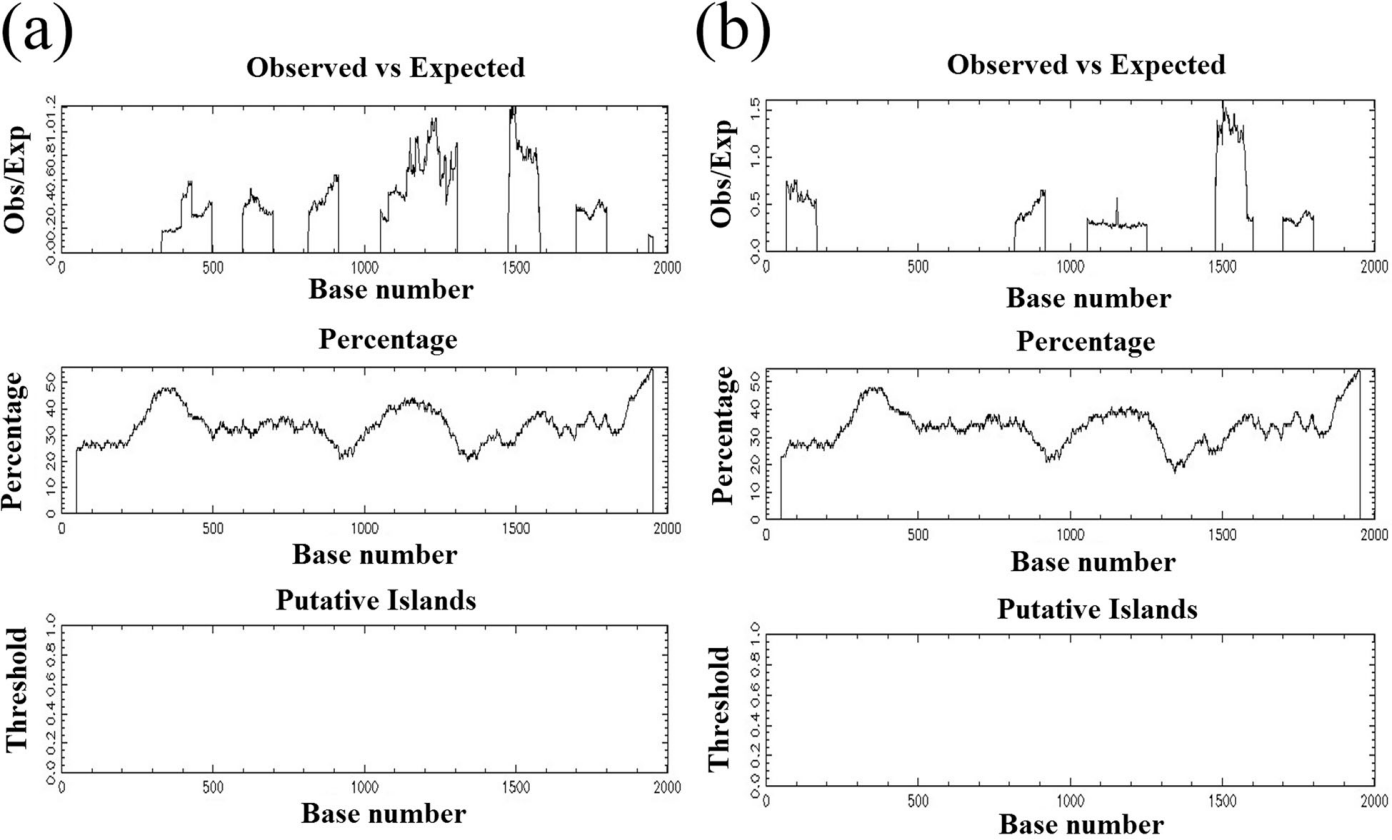
**a** Cow sequence length 3.0kb, CpG island prediction result, no CpG island was found, criteria used: island size > 100, GC percent greater > 50.0, Obs/Exp > 0.6. **b** Buffalo sequence length 3.0kb, CpG island prediction result, no CpG island were found, criteria used: island size > 100, GC percent greater > 50.0, Obs/Exp > 0.6

For the buffalo upstream from the starting ATG, we have also taken a 3.0-kb sequence to find out the CpG island, but not found in the sequence which is shown in Fig. 3. For further confirmation, we have used some other software tools for CpG island prediction and confirmation in cow and buffalo *AKT3* sequences, which show the same result with the previous software with no CpG island found in both species, as shown in Fig. 3.

### The predicted promoters of *AKT3* in cow and buffalo through bioinformatics

**Fig. 4:**
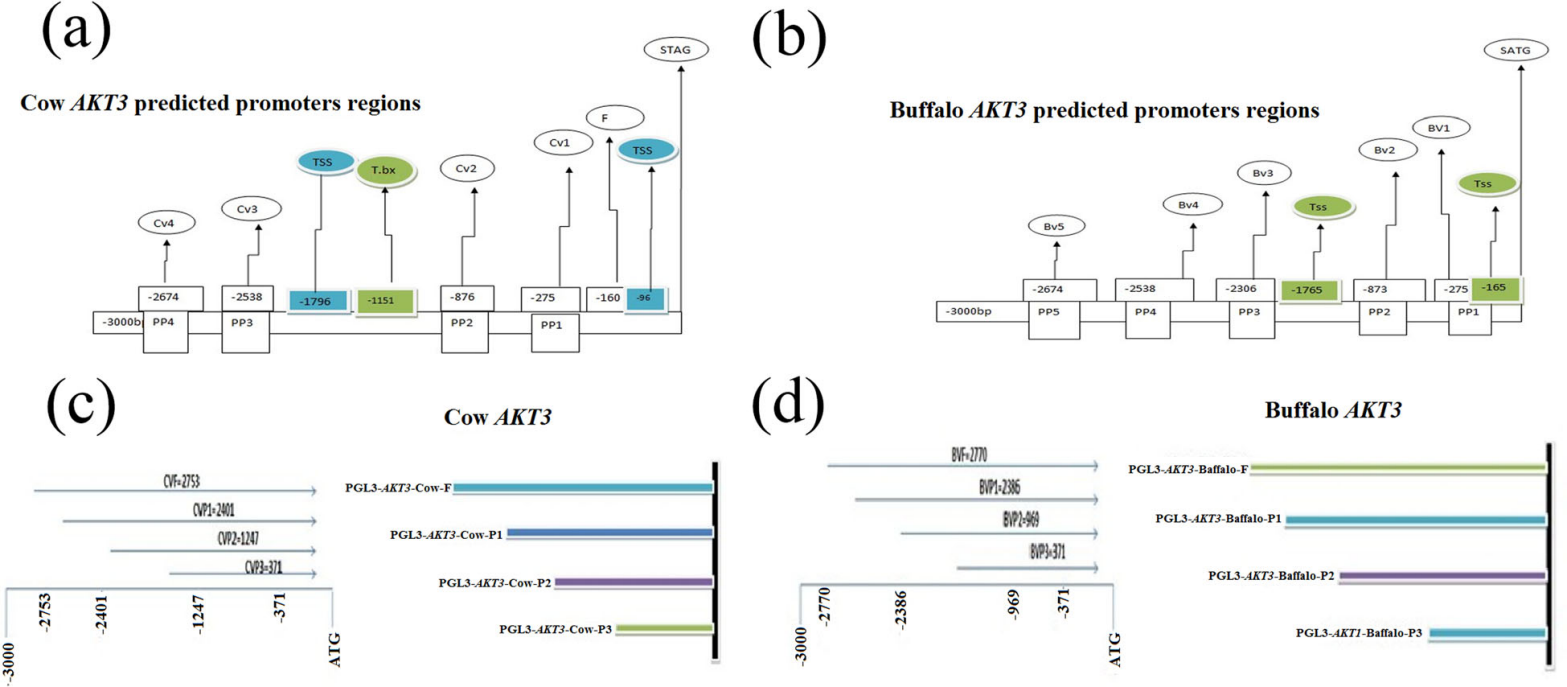
**a** The *AKT3* of cow predicted promoter position and distance from the starting ATG, with TSS and TATAA box. **b** The *AKT3* of buffalo predicted promoter position and distance from the starting ATG, with TSS. **c** Describes the amplified promoter regions from starting ATG in cow sequence. **d** Describes the amplified promoter regions from starting ATG in buffalo sequence

We have completely analyzed the upstream region from starting ATG of the cow and buffalo sequence of *AKT3* to design and prepare various primers and construct plasmid vectors based on upstream characterization in cow and buffalo. We diagrammatically described in cow and buffalo how these important consensuses occur as well promoters in the sequences. These are the basis to prepare and design various primers for these various regions of the predicted promoters and other sequences upstream of the gene in cows and buffalo. Based on the predicted location and position of the promoters and availability of the suitable primers, we have prepared four different reporter vectors to check and test the core promoter activity and expression in the cow and buffalo *AKT3* gene. Figure 4 describes the location and position of the predicted promoters, TSS, and TATA box in cow and buffalo sequences for *AKT3* (Fig. 4c). It is interesting to discuss that the ATG in cow sequence occurs in exon 1, while in buffalo sequence, it is situated in exon 2. Meanwhile, in 3000 bp of buffalo upstream sequence of ATG, just 5 promoter regions were predicted on various positions and locations, while in cow, there were four predicted promoter regions (Fig. 4d).

### Validation of *AKT3* promoters in cow and buffalo

The primers used in this experimental study are presented in Table 6. The KnpI and BgIII restriction sites and protective base were introduced to the 5’ end of the forward and reverse primers respectively. And, KpnI restriction sites and protective bases were introduced to the 5′ end of the forward primer, and BgIII restriction sites and protective bases were introduced to the 5′ end of the reverse primer.

**Table 6:** List of primers used in the experiment

No.	Primers	Temperature
**1**	ATTGGTACCCTCTGGTGGCTTTTTATCTTTGCT GGCAGATCTTCACAATGGTAACATCGCTCA	60.2
**2**	ATTGGTACC CGAAATGTAATCGTGCCCTC GGCAGATCTTCACAATGGTAACATCGCTCA	59.0
**3**	ATTGGTACCACCTTTTCCCACTTTCATCTTC GGCAGATCTTCACAATGGTAACATCGCTCA	60.0
**4**	ATTGGTACC CCTTTCACCTCAAACACCCAT GGCAGATCTTCACAATGGTAACATCGCTCA	59.2
**5**	GGCGGTACCCTTTCTTCAATACTTCCTTCCAG GGCAGATCTTCACAATGGTAACATCGCTCA	60.0
**6**	ATTGGTACCCAAGGCAGAGTAAGCATACATAAA GGCAGATCTTCACAATGGTAACATCGCTCA	60.2
**7**	ATTGGTACCATCTGCGAAATGTAATCGTGCC GGCAGATCTTCACAATGGTAACATCGCTCA	59.6
**8**	ATTGGTACCAAAGAATGTGGGCTTCAGAATCAGA GGCAGATCTTCACAATGGTAACATCGCTCA	60.4

### The functional proximal minimal promoter of *AKT3* gene

**Fig. 5:**
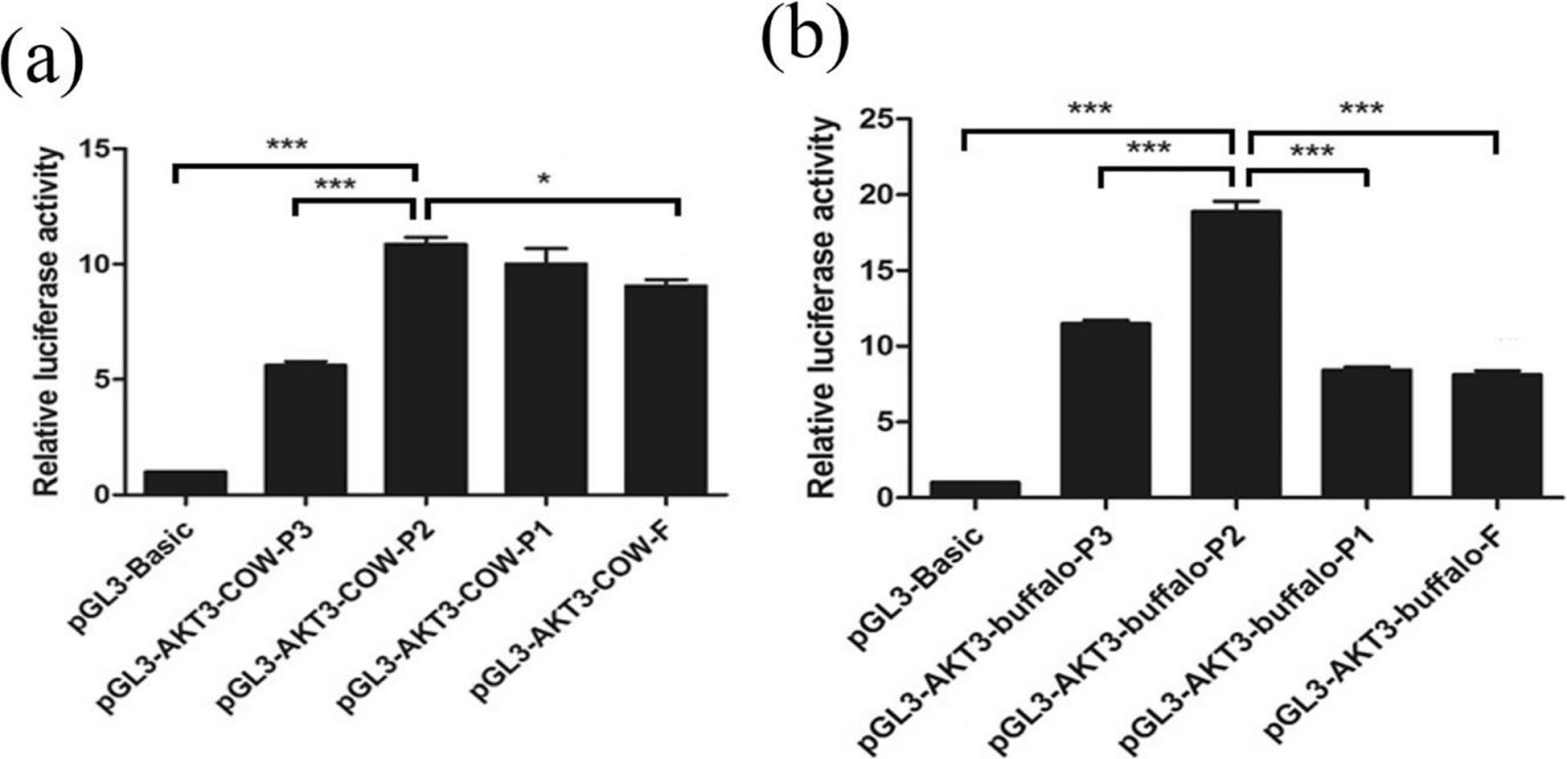
**a** The documentation and identification of *AKT3* core promoters in cow. These bars in 293T cells represent the comparative luciferase activities of the promoter fragments. **b** The certification, identification, and documentation of *AKT3* core promoters in buffalo. These bars in 293T cells represent the comparative luciferase activities of the promoter fragments. The values are represented as mean ± standard deviation. The standard deviations signified with the error bars respectively. To detect and clarify by using unpaired student’s *t*-test for significant differences as *P < 0.05, **P < 0.01, and ***P < 0.001. (*n* = 3)

To know and understand which predicted region is the minimum promoter sequence in the cow and buffalo of the *AKT3* gene, we have to compare the different predicted regions of the area to construct various regions between cow and buffalo. The expression and possessions of the reported construct alternate were investigated and estimated into the 293T cells after the transfection and the results are shown in Fig. 5. The different promoter construct reporters were tested and the results showing their activity are reported in the 293T cells in Fig. 5. The PGL3-*AKT3*-cow-p2 fragment promoter activity was significantly increased among the other different fragment promoters in cow *AKT3* in 293T cells, whereas in the buffalo *AKT3* in 293T cells, the promoter activity of the PGL3-*AKT3*-buffalo-P2 activity was higher as compared with the other adjacent corresponding promoters’ sequences in buffalo; these are shown in Fig. 5. The results of cow data indicate that the region upstream from − 371 to − 1247 bp was essential for *AKT3* in the cow sequence to maintain the promoter activity of the cow sequence, whereas in the buffalo data, results show that the fragment of the region from − 371 to − 969 was very essential and crucial for buffalo data sequence (Fig. 5b).

In Fig. 5a, the significant differences of the cow core promoter in 293T cells were presented. The four various fragments of the upstream reported construct vectors were compared with that of the control group and showed significant results while the buffalo data results are reported in Fig. 5a. For the buffalo data upstream of the promoter-reported construct vectors, four various fragments were prepared and compared with the control group.

### *AKT3* immune response of LPS-induced mastitis

The immune response of *AKT3* was observed for lipopolysaccharide (LPS) with various times intervals and duration [22]. LPS can disrupt the permeability of the blood-milk barrier by altering the expression of tight junction proteins [23]. For more information, LPS is a chemical component isolated from the cell wall of Gram-negative bacteria and is one of the important virulence factors for diseases caused by bacteria on bovine mammary glands [22]. Therefore, it was mined to observe and investigate the immunity response of *AKT3* with a time interval relationship with LPS. It will be helpful in future research plans directly concerned with mastitis. A list of the *AKT3* constructed primers which were used for the experiment is presented in Table 2.

**Fig. 6:**
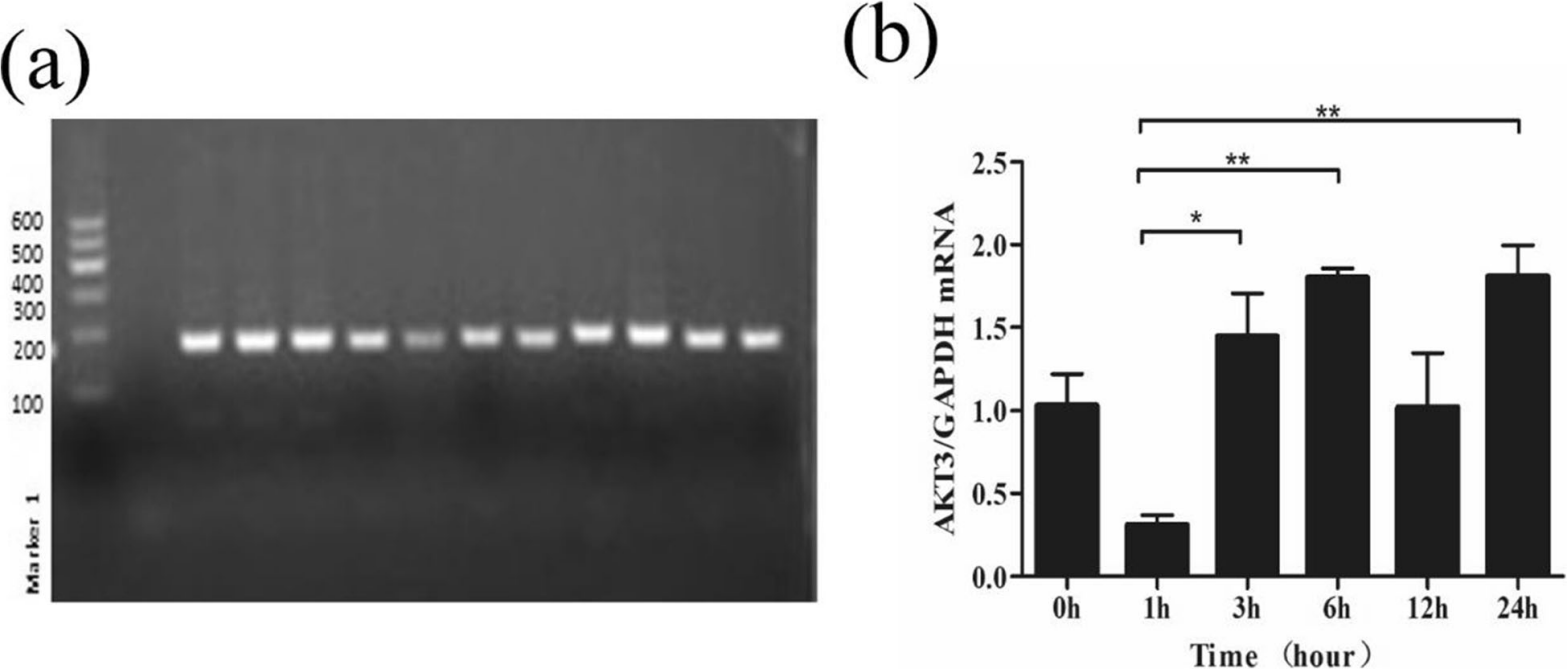
**a** The result of PCR product for mRNA of various primers. **b** The *AKT3* mRNA relative expression in bovine epithelium cell line was observed. The significantly different level of expression with time-dependent manner of LPS (10 ng/ml) treatment was found. The standard deviation (SD) is represented with error bars (*n* = 3). To detect and clarify by using unpaired student’s *t*-test for significant differences as *P < 0.05, **P < 0.01, and ***P < 0.001. (*n* = 3)

Moreover, the relative expression level of mRNA was checked for *AKT3* by using the bovine epithelium cell line (BEC). The bovine mammary epithelium cell line was used for the investigation of the relative expression of *AKT3* mRNA. Also, the mRNA relative expression was evaluated for *AKT3* with the time interval of 0, 1, 3, 6, 12, and 24 h. The higher levels of expression considerably were found in the mRNA of *AKT3* (*P* < 0.01) in response to 6 h of LPS (10 ng/ml) challenge. Meanwhile, the level of mRNA expression of *AKT3* with a dependent manner of immune response proved the significant difference with time of mRNA expression (Fig. 6b).

## Discussion

It has been reported that milk quality and quantity of cows and buffalos are influenced by various factors such as genetic profile, nutrition, infections, and environment. The molecular functions related to cow and buffalo milk production could be highly influenced by *AKT3* genes that are involved in kinase and ATP binding function [24]. Y Maetani et al. [25] reported that the ATP levels of fibroblast cells are significantly influenced by the AKT genes.

In this study, we focused on the promoter area of *AKT3* of cow and buffalo mammary glands. Mammary tissue is necessary for high milk production [15]. Comprehensive bioinformatic analyses for *AKT3* promoter activity sequence differences of the cow and buffalo could better highlight the immunity mechanisms, responses, and physiological differences of these two species. The results showed that in cows’ *AKT3* promoter region, sequences started from − 371 to − 876, while in buffaloes, it started from − 370 to − 969 crucial region of the promoter. Thus, the promoter activity is sustained by two core function promoter regions. This could play a key role in the inflammatory response and variation for susceptibility in buffalo and cows for mastitis or any other inflammatory disease [26]. Also, Jin et al. [27] mentioned that the differences in promoter region length could significantly affect the numbers of cell metabolic pathways that could make the differences in responses to the infections. Moreover, TSS/TATA was 2/7 for cows compared to 2/4 for buffalos in the core promoters. Zhang et al. [28] mentioned that TSS to TATA are playing an important role in RNA polymerase II transcription for the core promoter region. The core promoter region may contain various elements such as TATA. The core promoters exhibit significant diversity regarding the sequence and function of TATA. And the higher number of TATA in cows is increasing the transcription and therefore the sensitivity against pathogens compared to buffaloes. This could be from the increase in the TATA-binding protein subunit of the basal transcription factor that initiates transcription in conjunction with the initiator [29].

The *AKT3* mRNA expression results showed significant differences in the expression levels with a dependent manner of LPS stimulation in the bovine mammary epithelial cells. Yang et al. [30] reported that *Akt*3 is expressed during mammary gland development, and its expression is upregulated during lactation and is downregulated at the onset of involution. Moreover, the role of *Akt* promoters in regulating the lactation process was examined. And, its overexpression in the mammary glands resulted in the delay of involution and onset of apoptosis [30].

Despite functions specific differences in *AKT* isoforms, all the 3 subtypes are imminent entrant genes linked with milk production. Bionaz and Loor [31] reported that during lactation periods in cattle, the expression level of *AKT1* and *AKT3* was significantly increased. The synthesis of breast milk fat and cholesterol was affected by *the AKT* family.

**Fig. 7:**
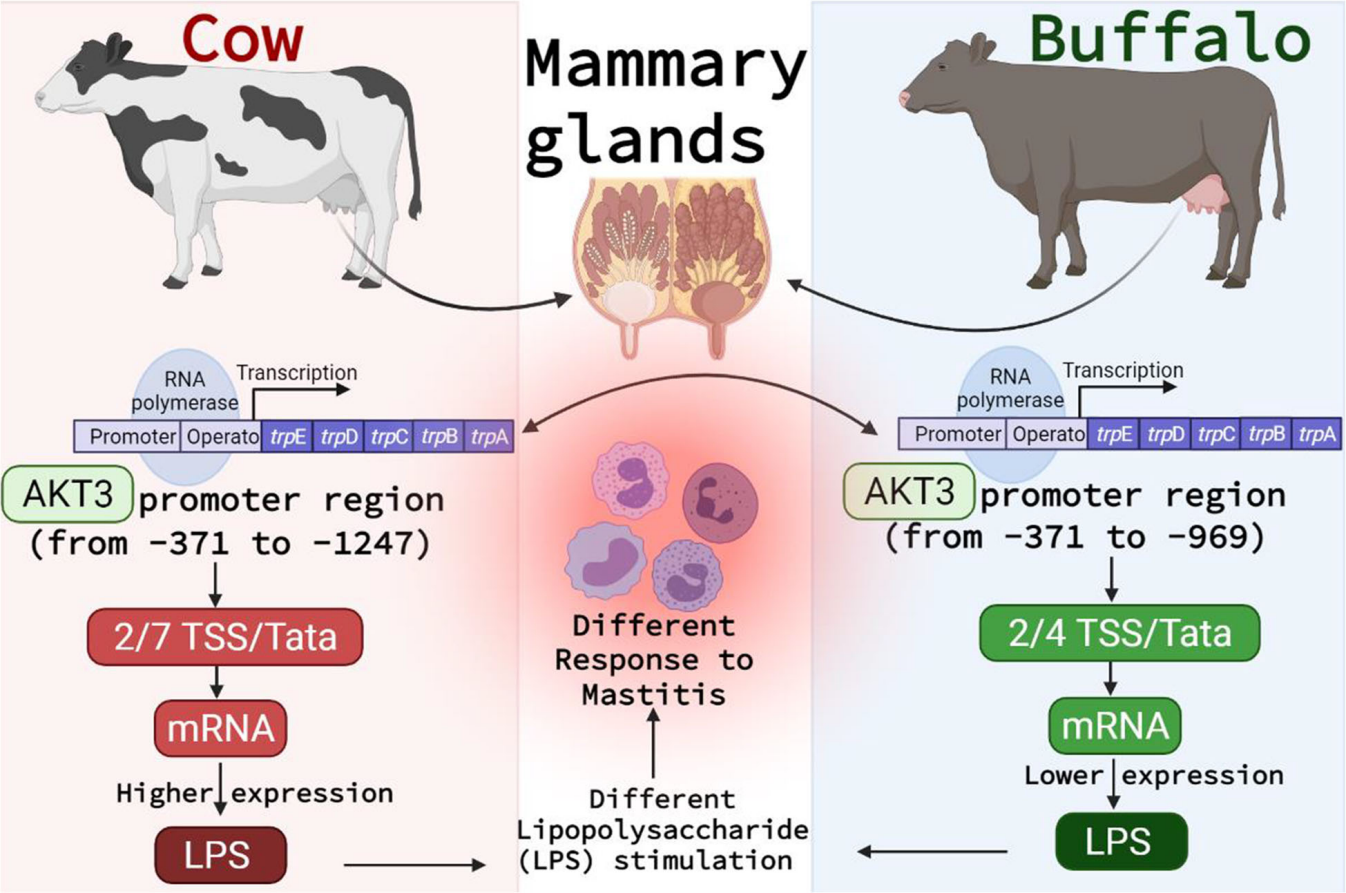
The different responses to mastitis between cow and buffalo based on the differences in *AKT3* gene promoters regions

The genetic differences among the promoters of the *AKT* family between cows and buffalos have significantly influenced not only their different biological processes like the produced milk composition but also their responses against infectious diseases like mastitis [15, 32]. The *AKT* family contributes to numerous disorders such as inflammation and ischemia [33]. Besides, the experimental autoimmune encephalomyelitis susceptibility is regulated by *AKT3*. In this study, results showed that the promoter region PGL3-*AKT3*-cow-P2 (− 371 to − 1247) in cow and PGL3-*AKT3*-buffalo-P2 (− 371 to 969) in buffalo was highly active as compared with the other regions in cow and buffalo (Fig. 5 and Fig. 7). The previous studies show that the isotope *AKT3* was involved in the different functions of the mammary gland cells. The prediction of promoter regions and consequent construction of reporter gene to check the activity of promoter region and analysis study were performed to understand the different functional phenomena of the promoter region. The activity of these different promoters was tested in 293 T cells and these regions showed that promoters’ activity was significantly increased in cows than buffaloes. This conserved region could be responsible for the pro-inflammatory signaling mechanism, and positive regulation of the gene.

Furthermore, the expression level of the various *AKT3* promoter regions reported that the distances from starting ATG of the *AKT3* in cow were − 225 to − 275, − 826 to − 876, − 2256 to − 2306, and − 2486 to − 2536, while for the buffalo, upstream CpG islands were not found in the sequence which is shown in Fig. 3. CpG island methylation was correlated with the possible transcription activity sites. Boulougouris et al. [34] mentioned that the CpG island is necessary for the conceivable activity sites for the transcription that is related to mammary gland lactation and mastitis disease.

The constructed *AKT3* promoters’ expressions were investigated using 293T cells. The results showed that buffaloes’ PGL3-*AKT3*-cow-p2 fragment promoters’ activity in 293T cells was significantly higher compared to cows. Also, the *akt3* promoter upstream region of cows was started from − 371 to − 1247 bp compared to buffaloes that started from − 371 to − 969 bp. Spangle et al. [35] mentioned that the differences in T cell functional responses could be from the differences in the *AKT3* promoter transcription that could lead to different pathways that correlated with a promoter-associated allele in cells.

For the immune response of *AKT3* and LPS, the conjugation between LPS and *AKT3* which is related to the immune response is important for predicting mastitis in its early stage of Gram-negative infections [36]. Therefore, the mastitis model based on the relationship between LPS and *AKT3* can provide a good platform for dairy cow mastitis research. Mastitis is a disease of inflammation that is concerned with immunity response and the immune response genes like *AKT3*. The higher levels of expression considerably were found in the mRNA of *AKT3* (*P* < 0.01) in response to 6 h of LPS (10 ng/ml) challenge. These results indicate that *AKT3* showed immunity response to LPS, and it could play an important role in mastitis, and mastitis is associated with inflammation of the udder [22].

This study’s limitations might be from the concerns of using RNA and DNA binding motifs, which are defined for a subset of some co-expressed genes only, and sometimes are great predictors of transcription factor binding [37]. Another limitation is that even when transcription factor binding is observed through experimental assays, this does not necessarily mean actual binding of the factor at the identified genomic location, nor automatically the regulation of the closest gene [38]. Finally, reproducibility still often remains a challenge for these approaches and the integration of the different online data from the databases like NCBI are not easy because of some more informative data from some sources than others. Therefore, in this study, we integrated both the animal and cell line experiments with the computational bioinformatics studies to face these limitations.

## Conclusion

The identification of sequences differences of *AKT3* and core promoter confirmation play an important key role in gene expression in cow and buffalo milk production and immune response against mastitis. The higher *AKT3* gene expression and transcription in cows than buffalos could have significant prevailing results between the two species. This could be one of the pro-inflammatory mechanisms responsible for the higher signaling phenomenon during pathogenic infection. This could be the reason that buffaloes are more resistant to mastitis disease than cows, while cow is more vulnerable as compared to buffalo. Continuing progress in genomics and systems biology technologies will aid in the understanding of the molecular and genetic bases of mastitis and other tumor heterogeneity and will enable us to identify more reliable diagnostic and prognostic biomarkers for metastatic and recurrent cattle diseases.

## Data Availability

All the data and their availability are included in the manuscript text, figures, tables, and supplementary materials. Also, all relevant raw data, will be freely available to any scientist wishing to use them for non-commercial purposes, without breaching.

## Abbreviations

AKT3: Serine/threonine kinase 3
BEC: Bovine epithelium cell line
cDNA: Complementary deoxyribonucleic acid
CDS: Gene-coding sequence
CDs: Coding regions
CNV: Copy number variants
DHI: Danhong injection
EDTA: Ethylenediaminetetraacetic acid
HBSS: Hanks’ balanced salt solution
KEGG: Kyoto Encyclopedia of Genes and Genomes
LPS: Lipopolysaccharide
NCBI: National Center for Biotechnology Information
PCR: Polymerase chain reaction
pGL3: Plasmid-basic-luciferase vector 3
SNPs: Single nucleotide polymorphisms
SREBP: Sterol regulatory element-binding protein
TLR: Toll-like receptor
TMR: Total mixed ration
TSS: Transcription start site
